# Psychological and motivational correlates of orthorexia nervosa: the role of perfectionism, emotion regulation, and health attitudes

**DOI:** 10.1007/s40519-026-01867-z

**Published:** 2026-05-21

**Authors:** Jessica Fanelli, Susanna Pardini, Anna Brytek-Matera, Caterina Novara

**Affiliations:** 1 Department of General Psychology, University of Padova, Padua, Italy; 2 Digital Health Research Unit, Centre for Health and Wellbeing, Fondazione Bruno Kessler, Trento, Italy; 3 Institute of Psychology, University of Wroclaw, Wroclaw, Poland

**Keywords:** Orthorexia nervosa, Eating disorders, Perfectionism, Emotion regulation, Health motivations, Obsessive–compulsive symptoms

## Abstract

**Background:**

Orthorexic tendencies are characterized by a rigid preoccupation with healthy eating and have been associated with eating disorder (ED) symptoms, perfectionism, and emotion regulation difficulties. However, the psychological and motivational correlates of the different dimensions of orthorexic symptomatology remain insufficiently understood.

**Methods:**

We conducted a cross-sectional, correlational study in a community sample of 300 Italian adults (mean age = 24.3 years, SD = 4.0). Participants completed an online survey including validated self-report measures of orthorexic tendencies (EHQ-21), ED symptomatology (EAT-26), obsessive–compulsive symptoms (OCI-R), perfectionism (MPS), emotion regulation difficulties (DERS), health and taste attitudes (HTAS), and eating motivations (EATMOT). Pearson’s correlations and multiple linear regression analyses were performed using both total scores and theoretically relevant subscales.

**Results:**

Orthorexic tendencies were positively associated with ED symptomatology, particularly dieting concerns. They were also positively associated with perfectionism, especially personal standards and concern over mistakes/doubts about actions. Associations with emotion regulation difficulties were generally weak at the total-score level, but the EHQ-21 Problems dimension showed positive correlations with non-acceptance of emotions, lack of confidence, and loss of control. Obsessive–compulsive symptoms were weakly and positively associated with orthorexic tendencies and did not emerge as significant predictors in regression models. Health-related variables showed the strongest pattern of associations: General Health Interest and Health Motivations were positively related to orthorexic dimensions, particularly Knowledge and Problems. In the regression model predicting the EHQ-21 total score, ED symptoms, perfectionism, and eating motivations emerged as significant predictors, explaining 44% of the variance.

**Conclusions:**

These findings suggest that orthorexic tendencies are more closely linked to ED-related features, perfectionism, and health-related motivations than to obsessive–compulsive symptoms. The results support a multidimensional view of orthorexic symptomatology and highlight the importance of distinguishing adaptive interest in healthy eating from more rigid and impairing orthorexic patterns.

## Background

Orthorexia nervosa (ON) refers to a maladaptive and rigid preoccupation with eating in a healthy way, characterized by the avoidance of foods perceived as unhealthy, excessive attention to food quality and composition, and adherence to inflexible dietary rules [1]. Such behaviors may lead to significant impairment in daily functioning and social relationships [2, 3]. Although ON is notformally recognized as a distinct diagnosis in the DSM-5 or ICD-11, it has attracted increasing research attentionbecause of its clinical resemblance to both eating disorders (EDs) and obsessive–compulsive disorder (OCD) [4–7]. The overlap between ON and EDs appears particularly relevant. ON shares several features with anorexia nervosa (AN), including restrictive eating patterns, rigid dietary rules, perfectionism, and a strong need for control [4, 8]. However, while AN is primarily characterized by concerns about weight, body shape, and thinness, orthorexic symptomatology is more specifically centered on beliefs about the health value or harmfulness of food [8, 9]. This distinction has led researchers to suggest that ON may represent a partially overlapping but conceptually distinct condition within the spectrum of disordered eating [8]. ON has also been compared with OCD, given the presence of intrusive food-related thoughts, contamination concerns, and ritualized food preparation behaviors [10]. Nevertheless, previous findings suggest that the association between ON and obsessive–compulsive symptoms may be more limited than initially assumed [11]. In addition, orthorexic behaviors are often experienced as ego-syntonic and coherent with a valued self-image, unlike the distressing and ego-dystonic experience more typically reported in OCD [12]. Taken together, these findings suggest that ON may share some obsessive features, while remaining more strongly connected to eating-related psychopathology. Among the psychological characteristics associated with ON, perfectionism has consistently emerged as one of the most relevant factors [13–17]. Individuals with elevated orthorexic tendencies often report high personal standards, excessive concern over mistakes, and rigid self-imposed rules regarding food selection and preparation. Perfectionistic strivings may therefore reinforce dietary rigidity and the pursuit of an idealized form of health through eating, contributing to the maintenance of orthorexic behaviors [18, 19]. Another construct of potential relevance is emotion regulation. Previous studies have shown that individuals with higher orthorexic tendencies may experience difficulties identifying, accepting, and regulating emotions, particularly under distress [20]. Rigid control over food intake may function as a maladaptive strategy to manage negative emotional states, similarly to what has been reported in other forms of eating psychopathology [21, 22]. At the same time, emotion regulation difficulties are considered a broad transdiagnostic vulnerability factor and therefore require further clarification in relation to orthorexic symptom dimensions specifically [23]. In addition to transdiagnostic psychological traits, recent literature has emphasized the importance of motivational and attitudinal factors in ON. In particular, orthorexic tendencies appear to be closely linked to health-related eating motivations, such as choosing foods primarily on the basis of perceived health benefits, as well as to a strong general interest in health [24–27]. By contrast, findings regarding other motives, such as ethical or environmental reasons for dietary restriction, are less consistent [25, 26, 28]. This suggests that ON may be better understood not only through general psychopathological traits, but also through the specific meanings and motivations individuals attach to their food choices. Against this background, the present study had two main aims: (1) to examine the associations between orthorexic tendencies and core psychopathological variables, including eating disorder symptomatology, obsessive–compulsive symptoms, perfectionism, and emotion regulation difficulties; and (2) to explore the role of health-related attitudes and eating motivations in orthorexic tendencies. Based on previous literature, we expected orthorexic tendencies to be more strongly associated with eating disorder symptomatology, perfectionism, and health-related motivations than with obsessive–compulsive symptoms, and to show positive associations with emotion regulation difficulties and health-related attitudes.

## Methodology

### Study design

This study employed a cross-sectional, observational design with correlational analyses. Data were collected through an online survey administered via Qualtrics. No intervention, randomization, or follow-up was performed. The present study is part of the Polish-Italian research project on perfectionism, emotional eating, eating motivation, and beliefs about losing control in orthorexia nervosa (MotivatiON study).

### Recruitment

**Fig. 1:**
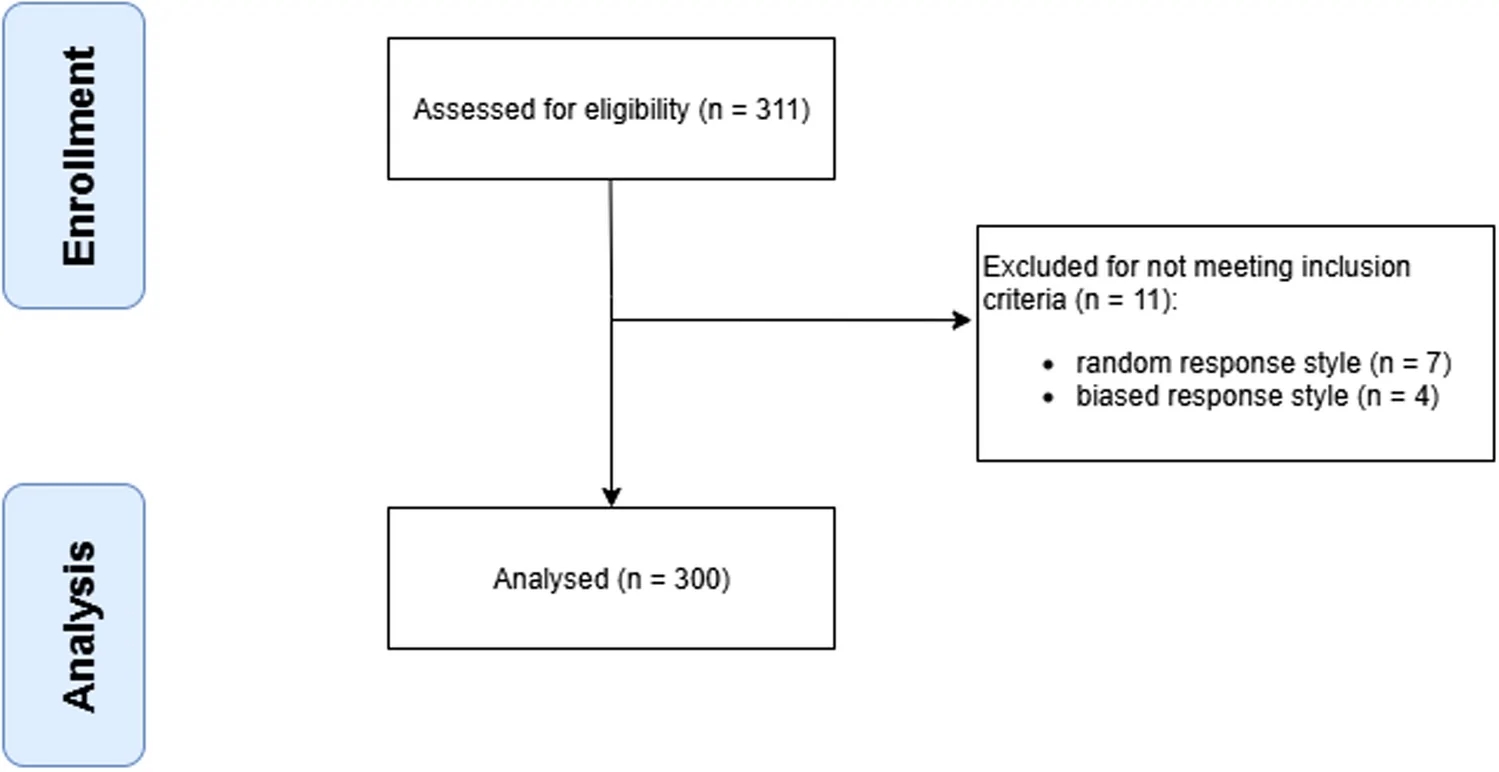
Flow diagram about the research phases

Participants were recruited in Italy using a non-probabilistic convenience sampling strategy, primarily through direct contacts, social media posts, university-related online groups (e.g., Facebook student groups), and messaging applications (e.g., WhatsApp). Recruitment was therefore mainly web-based and relied on voluntary participation. The sample was not designed to be representative of the Italian general population and may overrepresent younger, highly educated, and university-connected individuals. Due to these biases, socio-demographic features (e.g., age, years of education, gender, BMI) are considered in the data analysis process when interpreting the generalizability of the findings to the broader Italian population. Eligibility criteria included being at least 18 years old and being fluent in Italian. Exclusion criteria included self-reported severe psychiatric disorders, neurological conditions, and evidence of random or invalid response patterns. The final sample consisted of 300 participants. Figure 1 presents the main phases of the study and the reasons for participant exclusion.

### Ethical considerations

This study was conducted in accordance with the ethical standards of the Declaration of Helsinki. Ethical approval was obtained from the Ethics Committee of the University of Padua (code: 95-d; approval date: 23 October 2023). Before accessing the questionnaire, participants were informed about the aims of the study, the voluntary nature of participation, the confidentiality of their responses, and their right to withdraw at any time without consequences. Written informed consent was obtained electronically from all participants before participation. Data were stored securely and analyzed only in aggregate form. Given the observational design and the use of self-report questionnaires, the study involved minimal risk.

### Self-reported measures

Participants completed the following standardized self-reported measures: (1) Socio-demographic schedule (nationality, mother tongue, marital status, age, gender, height, weight, years of education, occupational status, psychological difficulties, dietary habits and medical conditions); (2) the *Eating Habits Questionnaire* (EHQ-21) [29, 30]: a 21-item measure designed to assess orthorexic tendencies, conceptualized as a pathological preoccupation with healthy eating. Items are rated on a 4 point Likert scale and divided into three subscales: *Knowledge* (beliefs and cognitions about healthy eating), *Problems* (negative consequences and impairments associated with rigid dietary patterns), and *Feelings* (emotional responses related to healthy eating). Although no universally established clinical cutoff is available, higher scores are generally interpreted as reflecting more problematic eating attitudes. In this study, internal consistency was excellent for the total score (α = 0.90); 3) the *Eating Attitudes Test* (EAT-26) [31, 32]: a 26-item screening questionnaire assessing symptoms and concerns characteristic of eating disorders. It includes three subscales: *Dieting* (concern about weight and body shape, avoidance of high-calories foods, desire to lose weight and fear of gaining weight), *Bulimia/Food Preoccupation* (intrusive thoughts about food, episodes of loss of control, compensatory behaviors including at a cognitive level), and *Oral Control* (control over food intake, perception of one’s own self-control over eating, perceived social pressure). Items are rated on a 6-point scale, and a total score ≥ 20 is commonly used as a cutoff indicating risk for clinically significant eating pathology. In this study, internal consistency ranged from acceptable to excellent (α = 0.60–0.91); (4) the *Obsessive–Compulsive Inventory-Revised* (OCI-R) [33, 34]: a 18-item self-report measure assessing obsessive–compulsive symptom dimensions, including *Washing*, *Checking*, *Ordering*, *Obsessing*, *Hoarding*, and *Neutralizing*. Items are rated on a 5-point Likert scale, with higher scores indicating greater symptom severity. A total score of 21 is often considered indicative of clinically relevant obsessive–compulsive symptoms. From a psychometric perspective, the OCI-R demonstrates good reliability and validity, and it is particularly used in both clinical and research settings. Internal consistency in this study was good to excellent (α = 0.72–0.90); (5); the *Multidimensional Perfectionism Scale* (MPS) [35, 36]: a 35-item scale among the most widely used for measuring perfectionism. The authors define perfectionism as the tendency to set high personal standards and to evaluate critically the failure to meet these standards. Thus, MPS assesses multiple dimensions of perfectionism including *Personal Standards*, *Concern over Mistakes*, *Doubts about Actions*, *Organization, Parental Expectations* and *Parental Criticism*. Items are rated on a 5-point scale and higher scores reflect higher levels of maladaptive and adaptive perfectionistic traits. No specific clinical cutoff is established, but elevated scores are associated with increased psychological vulnerability. Internal consistency in this study was excellent (α = 0.92); (6) the *Difficulties in Emotion Regulation Scale* (DERS) [37, 38]: a 33-item measure assessing clinically relevant difficulties in the regulation of negative emotions across six domains: *Non-acceptance of emotional responses*, *Difficulty in distraction*, *Lack of confidence*, *Impulse control difficultie*s, *Lack of emotional awareness*, and *Difficulty in recognizing emotions*. Items are rated on a 5-point scale and higher scores indicate greater difficulties in emotion regulation. While no universal cutoff is established, higher scores are associated with greater emotional dysregulation. Internal consistency in this study was excellent (α = 0.95); (7) the *Health and Taste Attitude Scales* (HTAS) [39, 40]: a 38-item instrument assessing attitudes toward food choices, particularly the relative importance of health and taste dimensions. It includes three subscales related to health (*General health interest*, *Light product interest*, *Natural product interest*) and three subscales related to taste (*Craving for sweet foods*, *Using food as a reward, Pleasure*). The Italian version of HTAS included the three original subscales related to health, *Using food as a reward* and two additional subscales regarding attitudes towards craving for sweet food based on their own experience (*Personal craving*) and attitudes towards other-people’s craving (*Other people craving*). Items are rated on a 7-point scale and higher scores reflect stronger endorsement of specific health or taste related attitudes. Internal consistency in this study ranged from acceptable to good (α = 0.65–0.93); (8) the *Eating Motivations Scale* (EATMOT) [41]: a 49-item scale aiming to capture different motivations underlying people’s food choices. The six subscales involve *Health motivations*, *Emotional motivations*, *Economic and availability motivations*, *Social and cultural motivations*, *Environmental and political motivations*, and *Marketing and commercial motivations*. Items are rated on a 5-point ordinal Likert scale and higher scores indicate stronger endorsement of specific motivational drivers. No clinical cutoff scores are defined. Internal consistency was acceptable overall (α = 0.70).

### Statistical analysis

All statistical analyses were conducted using R software [42]. Descriptive statistics (means, standard deviations, and frequencies) were computed to characterize the sample in terms of sex, native language, nationality, age, years of education, height, weight, marital status, occupational status, dietary habits, psychological difficulties and medical conditions. In order to ensure the reliability of the results, the internal consistency of all psychometric instruments was evaluated using Cronbach’s alpha coefficients. Accordingly to the first aim of this study, Pearson’s correlation coefficients were calculated to examine bivariate associations between orthorexia nervosa and the psychological and motivational variables of interest. To further explore the role of health-related attitudes and eating motivations in ON, multiple linear regression analyses were performed. A portion of the regression analyses was conducted to examine the role of broad psychological constructs—namely perfectionism, difficulties in emotion regulation, eating disorder symptomatology, obsessive–compulsive symptomatology, health-related attitudes, and eating motivations—in orthorexia nervosa and its various dimensions. A further set of analyses was aimed at investigating the contribution of specific aspects of these constructs in predicting orthorexia-related beliefs, negative consequences, and emotional experiences. All the tested models included age and years of education as covariates in order to account for their potential effects. Moreover, all tests were two-tailed, and the level of statistical significance was set at p < 0.05. Effect sizes were interpreted according to Cohen’s guidelines (small = 0.10, medium = 0.30, large = 0.50) [43].

### Participants

The main characteristics of the sample are reported in Table 1. A total of 300 Italian adults participated in the study, of whom 289 (96%) identified as Italian. The mean age was 24.27 years (SD = 4.02), with an average of 16.55 years of education (SD = 2.36). The sample had a mean weight of 64.06 kg (SD = 12.72) and mean height of 169.20 cm (SD = 9.18). The body mass index (BMI) was calculated on the reported weight and height, resulting in an average BMI of 22.26 (SD = 3.36). No significant gender differences were observed. Regarding occupational status, 188 participants (63%) were students, 66 (22%) were employed full-time or part-time, 22 (7%) were unemployed or in precarious employment, and 24 (8%) reported other unspecified occupations. Psychology was the most common field of study or work (n = 106; 35%). Fifty-three participants (18%) reported experiencing psychological difficulties, most frequently anxiety (n = 21; 7%). Seven participants (2%) reported a current or past eating disorder diagnosis: bulimia (n = 1), binge-eating disorder (n = 1), unspecified eating disorder (n = 1), and comorbidities including at least one eating disorder (n = 4). Additional reported conditions (8%) included depression, bipolar disorder, obsessive–compulsive disorder, post-traumatic stress disorder, and other comorbid mental health issues. Among those reporting psychological difficulties, the majority (n = 48; 91%) indicated that symptoms had persisted for at least one to five years. In terms of dietary habits, 170 participants (57%) did not follow any specific nutritional pattern, 79 (26%) adhered to a Mediterranean diet, and 30 (10%) followed a vegetarian diet. More than half of the sample (n = 159; 53%) reported avoiding certain types of food, most commonly sugary drinks and sweets (n = 29; 10%), dairy products (n = 16; 5%), fatty foods (n = 16; 5%), meat (n = 16; 5%), both meat and fish (n = 15; 5%), and processed foods (n = 12; 4%). Finally, 49 participants (16%) reported suffering from medical conditions, including asthma, thyroid dysfunctions, diabetes, irritable bowel syndrome, gastrointestinal diseases, food-related allergies or intolerances, and gynecological disorders.

**Table 1:** Demographic and clinical characteristics of the sample (N = 300)

Variable	n (%)
Gender	
Female	216 (72%)
Male	84 (28%)
Italian native language	
Yes	290 (96.7%)
No	10 (3.3%)
Marital status	
Single/in a relationship, not cohabiting	259 (86.3%)
Married/cohabiting	37 (12.3%)
Separated/divorced	4 (1.3%)
BMI	
Low	26 (8.7%)
Norm	227 (75.7%)
High	46 (15.3%)

*BMI* Body Mass Index [BMI categories were defined according to World Health Organization criteria: low (BMI < 18.5 kg/m^2^), normal (BMI = 18.5–24.99 kg/m^2^), and high (BMI ≥ 25 kg/m^2^)] [44]

## Results

### Correlational analyses

As shown in Table 2, the overall level of orthorexia nervosa symptoms (EHQ-21 total score) was positively and strongly correlated with the overall level of eating disorder symptoms (EAT-26 total score) and showed positive medium-to-strong correlations with all dimensions of eating disorder symptomatology, namely dieting, bulimic symptoms/food preoccupation, and oral control. Among the orthorexia dimensions, knowledge about healthy eating (EHQ-21 Knowledge) was positively and moderately associated with dieting (EAT-26 Dieting), whereas problems associated with healthy eating (EHQ-21 Problems) were positively and strongly related to overall eating disorder symptoms, dieting, and bulimic symptoms/food preoccupation, and positively and moderately related to oral control. The positive feelings associated with healthy eating (EHQ-21 Feelings) dimension was positively and moderately associated with the overall level of eating disorder symptoms and with all three specific dimensions. As shown in Table 2, the overall level of orthorexia nervosa symptoms was positively and moderately correlated with overall perfectionism (MPS total score), as well as with the perfectionism dimensions reflecting high personal standards (MPS Personal Standards) and concern over mistakes/doubts about actions (MPS Concern over Mistakes and Doubts about Actions). More specifically, the knowledge dimension was positively associated with personal standards, the problems dimension was positively associated with concern over mistakes and doubts about actions, and the feelings dimension showed the strongest positive associations with both of these perfectionism-related traits. Correlations with eating motivations (EATMOT) are reported in Table 2. The overall level of orthorexia nervosa symptoms was positively and moderately associated with both overall eating motivations and, in particular, with health-related motivations for food choice. It also showed a weak positive association with emotional motivations and environmental/political motivations, but a weak negative association with marketing and commercial motivations. The knowledge about healthy eating dimension showed its strongest positive correlation with health-related motivations, was positively associated with environmental/political motivations, and was negatively associated with marketing and commercial motivations, while no significant association emerged with emotional motivations. The problems associated with healthy eating dimension was positively associated with overall eating motivations, health-related motivations, emotional motivations, and environmental/political motivations, whereas its association with marketing and commercial motivations was not significant. The positive feelings associated with healthy eating dimension was positively associated with overall eating motivations, health-related motivations, emotional motivations, and environmental/political motivations, and negatively associated with marketing and commercial motivations. As shown in Table 2, correlations with health- and taste-related attitudes toward food (HTAS) showed a mixed pattern. The overall level of orthorexia nervosa symptoms was positively and moderately associated with the overall index of health- and taste-related attitudes and positively and strongly associated with general interest in healthy eating. It was also positively associated with interest in natural products, but negatively associated with personal craving and using food as a reward, while associations with light product interest and other people’s craving were not significant. A similar pattern emerged across orthorexia dimensions. In particular, all three dimensions were positively associated with general interest in healthy eating, whereas knowledge about healthy eating and problems associated with healthy eating were negatively associated with using food as a reward. In addition, knowledge was negatively associated with personal craving, whereas feelings showed no significant association with this dimension. Regarding difficulties in emotion regulation (DERS) (Table 2), the overall level of orthorexia nervosa symptoms showed positive weak-to-moderate correlations with most emotion regulation difficulties, including overall emotion regulation difficulties, non-acceptance of emotional responses, difficulty in distraction, lack of confidence, lack of control, and difficulty in recognizing emotions, whereas the association with reduced self-awareness was not significant. The knowledge about healthy eating dimension was largely unrelated to emotion regulation difficulties, but showed a negative association with reduced self-awareness. In contrast, the problems associated with healthy eating dimension was positively and moderately correlated with overall emotion regulation difficulties and with most of their specific dimensions, particularly lack of confidence, lack of control, and non-acceptance of emotional responses. The positive feelings associated with healthy eating dimension was also positively associated with several emotion regulation difficulties, including overall difficulties, non-acceptance, difficulty in distraction, lack of confidence, and lack of control, and negatively associated with reduced self-awareness. Finally, correlations with obsessive–compulsive symptoms (OCI-R) were generally weak and mixed in direction, as reported in Table 2. The overall level of orthorexia nervosa symptoms was positively associated with overall obsessive–compulsive symptoms and specifically with ordering, washing, and obsessing, whereas associations with hoarding, checking, and mental neutralizing were not significant. The knowledge about healthy eating dimension showed no significant associations with obsessive–compulsive symptoms overall and was negatively associated only with hoarding and mental neutralizing, although these associations were not significant. By contrast, the problems associated with healthy eating dimension was positively associated with overall obsessive–compulsive symptoms and with all obsessive–compulsive dimensions. The positive feelings associated with healthy eating dimension was positively associated with overall obsessive–compulsive symptoms, as well as with ordering and obsessing, while the remaining associations were weak and not statistically significant.

**Table 2:** Pearson’s correlations between the EHQ-21 and the EAT-26, the MPS, the EATMOT, the HTAS, the DERS, the OCI-R (N = 300)

Variable	EHQ-21 total	EHQ-21 knowledge	EHQ-21 problems	EHQ-21 feelings
EAT-26-Total	0.60^***^	0.28^***^	0.66^***^	0.44^***^
EAT-26-D	0.62^***^	0.30^***^	0.66^***^	0.44^***^
EAT-26-B/FP	0.44^***^	0.15^**^	0.50^***^	0.34^***^
EAT-26-OC	0.42^***^	0.19^***^	0.46^***^	0.32^***^
MPS Total	0.35^***^	0.22^***^	0.29^***^	0.37^***^
MPS-CDM	0.31^***^	0.12^*^	0.30^***^	0.32^***^
MPS-O	0.12^*^	0.19^***^	0.03	0.14^**^
MPS-PS	0.36^***^	0.33^***^	0.25^***^	0.36^***^
MPS-PEPC	0.19^***^	0.07	0.19^***^	0.22^***^
EATMOT-Total	0.36^***^	0.28^***^	0.32^***^	0.26^***^
EATMOT-HM	0.49^***^	0.53^***^	0.38^***^	0.29^***^
EATMOT-EM	0.14^**^	0.01	0.15^**^	0.20^***^
EATMOT-EP	0.21^***^	0.25^***^	0.14^*^	0.15^**^
EATMOT-MC	−0.15^**^	−0.26^***^	−0.02	−0.15^**^
HTAS-Total	0.31^***^	0.23^***^	0.27^***^	0.25^***^
HTAS-GHI	0.68^***^	0.64^***^	0.58^***^	0.43^***^
HTAS-LPI	0.001	−0.07	0.06	−0.04
HTAS-NPI	0.26^***^	0.28^***^	0.21^***^	0.16^**^
HTAS-PC	−0.11^*^	−0.15^**^	−0.11	0.01
HTAS-OPC	0.02	−0.1	0.07	0.07
HTAS-UFR	−0.21^***^	−0.17^**^	−0.23^***^	−0.04
DERS-Total	0.27^***^	−0.01	0.36^***^	0.21^***^
DERS-NER	0.22^***^	−0.02	0.30^***^	0.17^**^
DERS-DD	0.22^***^	0.03	0.24^***^	0.26^***^
DERS-LC	0.28^***^	0.01	0.38^***^	0.20^***^
DERS-ICD	0.28^***^	0.05	0.33^***^	0.24^***^
DERS-DI	0.16^**^	−0.07	0.25^***^	0.11
DERS-RSA	−0.02	−0.17^**^	0.11	−0.13^*^
OCI-R-Total	0.22^***^	0.01	0.27^***^	0.20^***^
OCI-R-H	0.07	−0.06	0.12^*^	0.07
OCI-R–C	0.11	−0.01	0.15^**^	0.09
OCI-R-Or	0.20^***^	0.07	0.20^***^	0.21^***^
OCI-R-MN	0.08	−0.07	0.16^**^	0.01
OCI-R-W	0.18^***^	0.05	0.22^***^	0.11
OCI-R-Ob	0.27^***^	0.05	0.29^***^	0.28^***^

*D* Dieting, *B/FP* bulimia/food preoccupation, *OC* oral control, *CMD* concern over mistakes and doubts about actions, *O* organization, *PS* personal standards, *PEPC* parental expectations and parental criticism, *HM* health motivations, *EM* emotional motivations, *EP* environment and politics motivations, *MC* marketing and commercial motivations, *GHI* general health interest, *LPI* light product interest, *NPI* natural product interest, *PC* personal craving, *OPC* other people’s craving, *UFR* using food as reward, *NER* non-acceptance of emotional responses, *DD* difficulty in distraction, *LC* lack of confidence, *ICD* lack of control, *DI* difficulty in recognizing emotions, *RSA* reduced self-awareness, *H* hoarding, *C* checking, *O* Ordering, *MN* mental neutralizing, *W* washing, *Ob* obsessing

*p-value < 0.05

**p-value < 0.01

***p-value < 0.001

### Multiple linear regression analyses

A multiple linear regression model was estimated with the overall level of orthorexia nervosa symptoms (EHQ-21 total score) as the dependent variable and the overall levels of eating disorder symptoms (EAT-26 total score), obsessive–compulsive symptoms (OCI-R total score), difficulties in emotion regulation (DERS total score), health- and taste-related attitudes toward food (HTAS total score), perfectionism (MPS total score), and eating motivations (EATMOT total score) as predictors. As shown in Table 3, eating disorder symptoms, perfectionism, and eating motivations emerged as significant predictors of orthorexic tendencies, accounting for 44% of the variance (R^2^ = 0.44; F(8,288) = 27.82; p < 0.001). Additional regression models were conducted separately for each dimension of orthorexia nervosa symptoms. First, the total scores of all predictor variables were entered simultaneously (Table 3). Second, in line with our hypotheses, further models were tested using specific dimensions of theoretical interest, namely dieting, general interest in healthy eating, high personal standards, and health-related motivations for food choice (Tables 4). For the knowledge about healthy eating dimension, significant predictors included eating disorder symptoms, difficulties in emotion regulation, perfectionism, and eating motivations. Together, these variables explained 20% of the variance (R^2^ = 0.20; F(8,288) = 8.92; p < 0.001). When looking at the specific constructs associated with this dimension, interest in general health, high personal standards and health-related motivations for food choice were the significant predictors, explaining 47% of the variance (R^2^ = 0.47; F(6,290) = 42.82; p < 0.001). For the problems associated with healthy eating dimension, eating disorder symptoms and eating motivations were the strongest predictors, accounting for 47% of the variance (R^2^ = 0.47; F(8,288) = 32; p < 0.001). Dieting and interest in general health were the specific variables predicting this dimension, explaining 55% of the variance (R^2^ = 0.55; F(6,290) = 59.89; p < 0.001). About the feelings associated with healthy eating dimension, eating disorder symptoms and perfectionism emerged as significant predictors, explaining 27% of the variance (R^2^ = 0.27; F(8,288) = 13.22; p < 0.001). High personal standards, dieting and interest in general health were the significant specific predictors, accounting for 31% of the variance (R^2^ = 0.31; F(6,290) = 21.74; p < 0.001). Age and years of education were included as covariates in order to account for their confounding effects. Education showed a significant effect (β =−0.09; p < 0.05) in predicting problems associated with orthorexia nervosa when specific predictors were considered in the model (Table 4), but not overall.

**Table 3:** Multiple regression model predicting the EHQ-21 scores with age and years of education as covariates

Predictor	B	β (standardized)	SE	T
EHQ-21 total				
EAT-26 total	0.56	0.53	0.05	10.17^***^
OCI-R total	0.02	0.02	0.05	0.35
DERS total	−0.04	−0.09	0.03	−1.50
HTAS total	0.03	0.05	0.03	0.97
MPS total	0.06	0.13	0.02	2.60^**^
EATMOT total	0.19	0.19	0.05	3.60^***^
Age	0.09	0.03	0.12	0.69
Years of education	0.01	0.001	0.20	0.02
EHQ-21 knowledge				
EAT-26 total	0.09	0.25	0.02	4.08^***^
OCI-R total	−0.01	−0.02	0.02	−0.23
DERS total	−0.04	−0.26	0.01	−3.59^***^
HTAS total	0.01	0.05	0.01	0.83
MPS total	0.03	0.19	0.01	3.06^**^
EATMOT total	0.08	0.23	0.02	3.66^***^
Age	−0.003	−0.004	0.05	−0.07
Years of education	0.12	0.08	0.08	1.42
EHQ-21 problems				
EAT-26-total	0.37	0.59	0.03	11.63^***^
OCI-R total	0.01	0.03	0.03	0.45
DERS total	0.01	0.04	0.02	0.73
HTAS total	0.01	0.03	0.02	0.49
MPS total	0.004	0.01	0.01	0.26
EATMOT total	0.09	0.14	0.03	2.62^**^
Age	0.09	0.06	0.07	1.38
Years of education	−0.14	−0.05	0.12	−1.21
EHQ-21 feelings				
EAT-26 total	0.09	0.34	0.02	5.65***
OCI-R total	0.01	0.04	0.01	0.60
DERS total	−0.01	−0.11	0.01	−1.54
HTAS total	0.01	0.07	0.01	1.12
MPS total	0.03	0.23	0.01	3.98***
EATMOT total	0.03	0.12	0.02	1.91
Age	−0.01	−0.02	0.04	−0.28
Years of education	0.03	0.03	0.06	0.50

*SE* standard error

*p-value < 0.05

**p-value < 0.01

***p-value < 0.001

**Table 4:** Regression model predicting the EHQ-21 subscales (selected predictors) with age and years of education as covariates

Predictor	B	β	SE	t
EHQ-21 knowledge				
EAT-26 dieting	0.01	0.009	0.03	0.19
HTAS general health	1.58	0.44	0.23	6.80^***^
MPS personal standard	0.11	0.18	0.03	4.02^***^
EATMOT health motivations	0.14	0.22	0.04	3.77^***^
Age	−0.04	−0.04	0.04	−0.92
Years of education	0.09	0.06	0.07	1.42
EHQ-21 problems				
EAT-26 dieting	0.48	0.49	0.04	10.84***
HTAS general health	2	0.32	0.38	5.32***
MPS personal standard	0.05	0.05	0.04	1.12
EATMOT health motivations	0.07	0.06	0.06	1.10
Age	0.05	0.03	0.06	0.80
Years of education	−0.24	−0.09	0.11	−2.27*
EHQ-21 feelings				
EAT-26 dieting	0.12	0.27	0.02	4.79^***^
HTAS general health	0.53	0.19	0.20	2.62^**^
MPS personal standard	0.10	0.22	0.02	4.34^***^
EATMOT health motivations	0.04	0.09	0.03	1.32
Age	-0.02	-0.03	0.03	-0.63
Years of education	0.003	0.003	0.06	0.06

*SE* standard error

*p-value < 0.05

**p-value < 0.01

***p-value < 0.001

## Discussion

The present study investigated the relationships between orthorexia nervosa and other psychopathological constructs, our findings confirmed a strong association between ON and ED characteristics, and ED symptomatology frequently emerged as significant predictors in regression models. In line with the growing body of literature showing robust associations between orthorexic tendencies and eating-disorder psychopathology in both clinical and non-clinical samples, our findings further confirm the relevance of ED-related symptoms in ON [45–47]. Among ED features, concerns about body weight and shape, were the strongest correlates of ON, except for its core beliefs. This suggests that while ON and ED share problematic behaviors—such as rigid food avoidance, dietary restraint, and compulsive exercise [48], their underlying beliefs differ. Taken together, these findings suggest that orthorexic tendencies are closely linked to restrictive eating attitudes and weight-control behaviors, which are central features of traditional ED psychopathology [26, 49]. Despite these similarities, the present findings also suggest differences in the underlying cognitive motivations. This is in line with theoretical accounts proposing that, unlike eating disorders such as anorexia nervosa or bulimia nervosa, orthorexic cognitions are primarily driven by an excessive preoccupation with health and well-being rather than by fears related to weight gain or body shape [8, 50, 51]. As hypothesized, ON showed a modest association with obsessive–compulsive tendencies, particularly with the Problems and Feelings dimensions. However, obsessive–compulsive symptoms did not significantly predict ON in the regression analyses [13]. These findings suggest that, although orthorexic symptomatology may include repetitive thoughts and ritualized behaviors resembling obsessive–compulsive manifestations [52, 53], the phenomenological and motivational foundations of the two conditions differ substantially [12, 13]. This distinction is clinically relevant, as repetitive behaviors in ON may be more accurately conceptualized as goal-directed and value-consistent rather than compulsive in nature [54].

Consistent with predictions, ON was strongly associated with perfectionism, particularly with high personal standards, and to a lesser extent with concerns over mistakes and doubts about actions. This finding aligns with prior research, which demonstrates that both perfectionistic strivings and concerns are associated with orthorexic tendencies [16, 18]. Individuals with ON may channel perfectionistic standards into rigid adherence to health-promoting diets, experiencing satisfaction when dietary rules are maintained and distress when perceived standards are not met. The tendency to show negative reactions to mistakes, to interpret mistakes as a failure, and to worry that tasks have not been completed properly may especially arise when a disruption between a perfectionistic standard and reality occurs [55, 56]. Perfectionism may indirectly exacerbate orthorexic symptomatology by fostering a self-concept and sense of personal worth predominantly centered on health [57]. In this regard, research suggests that orthorexia nervosa is often embedded within a broader health-oriented lifestyle that may be pursued rigidly [58, 59] and may foster feelings of superiority based on eating habits [3, 60]. The present findings offer a more articulated view of the role of emotion regulation in ON. Consistent with previous literature, ON was associated with difficulties in emotion regulation, particularly in terms of non-acceptance of emotional responses, reduced behavioral control under distress, and lower emotional confidence. These patterns align with prior evidence identifying non-acceptance and impulse-control difficulties as relevant correlates and predictors of orthorexic tendencies [61]. However, when considered within a multivariate framework, emotion-regulation difficulties did not emerge as significant predictors of overall ON, with the exception of the cognitive dimension. This result is consistent with previous findings [20, 62], suggesting that the contribution of emotional dysregulation to ON may diminish once eating-disorder symptomatology is taken into account. A more nuanced pattern emerged when examining specific dimensions of ON. Emotion-regulation difficulties significantly predicted the knowledge about healthy eating dimension, but not the more impairing components (i.e., problems and feelings associated with healthy eating). This suggests that emotional processes may be more closely linked to the cognitive engagement with healthy eating rather than to its dysfunctional manifestations. At the same time, ON cognitions showed a negative association with overall emotion-regulation difficulties, primarily driven by an inverse relationship with lack of emotional awareness. In line with previous research [61], this finding indicates that individuals endorsing stronger orthorexic beliefs may exhibit greater attention to internal emotional states. Importantly, heightened emotional awareness does not necessarily translate into more adaptive regulatory strategies. Taken together, these findings point to a differentiated role of emotion regulation in ON: while increased emotional awareness may characterize the cognitive and interest-based aspects of orthorexia, maladaptive regulatory patterns, such as emotional non-acceptance and reduced behavioral control, may be more relevant for its clinically impairing features, although their predictive role appears secondary to that of eating-disorder symptoms [61]. Overall, these results highlight the complexity of emotional processes in ON and underscore the need for further research to clarify their specific contribution to both adaptive and maladaptive facets of the condition. Health motivations emerged as significant predictors of ON, especially in relation to orthorexic beliefs. This finding is consistent with the limited literature emphasizing the centrality of health-related motives in ON [25, 26]. Perfectionism may indirectly exacerbate orthorexic symptomatology by fostering a self-concept and sense of personal worth predominantly centered on health [57]. In this regard, research suggests that ON is often embedded within a broader health-oriented lifestyle that may be pursued rigidly [58, 59] and may foster feelings of superiority based on eating habits [3, 60]. Notably, the present findings are among the first to highlight the specific role of health-related food-choice motivations in ON. Within this framework, ON may be conceptualized not merely as a pattern of dietary restriction, but as a cognitive–motivational system grounded in the pursuit of perceived optimal health, purity, and bodily efficiency, which progressively becomes rigid and self-reinforcing. Our results also highlighted the specific and unique role of interest in general health, as assessed by the Health and Taste Attitudes Scale. Notably, it emerged as the most consistent predictor across all ON dimensions, whereas the overall attitudes did not show a significant level of association. Once again, this suggests that ON may be distinguished from other psychopathologies by the salience of health-related attitudes, rather than by concerns about food, weight or body image, reflecting an intensified form of health investment that becomes cognitively dominant and behaviorally guiding [1]. However, to the best of our knowledge, few studies have systematically investigated the role of health attitudes in orthorexia [27, 63] [e.g. 64–66]. Therefore, further research is needed for this purpose. Taken together, these findings indicate that ON shares essential features with EDs and perfectionism, while its overlap with OCD is limited. Health-related motivations and general interest in health appear to play a central role in shaping orthorexic tendencies, and difficulties in emotion regulation further contribute to the maladaptive aspects of the condition. These results underscore the importance of considering both transdiagnostic traits and specific health-oriented motivations when conceptualizing ON, with implications for prevention and treatment.

### Clinical implications and limitations

The present findings have several clinical implications. First, the strong association between ON and eating disorder symptomatology underscores the need for clinicians to carefully assess restrictive eating behaviors that may initially appear health-oriented but carry a significant risk of impairment. Identifying perfectionistic strivings and rigid health-related beliefs may be particularly useful for differentiating between adaptive health consciousness and pathological orthorexia. Furthermore, the role of emotion regulation difficulties suggests that therapeutic approaches targeting emotional awareness, acceptance, and impulse control may be beneficial for individuals with orthorexic tendencies. Finally, the centrality of health motivations underscores the importance of addressing dysfunctional health beliefs and educating patients on adopting flexible, balanced approaches to nutrition. Several limitations should be acknowledged. The cross-sectional design prevents causal inferences, and the exclusive use of self-report measures may be affected by response biases. The sample, although relatively large, was predominantly composed of young, highly educated individuals, which may limit generalizability to other populations. Additionally, the reliance on convenience sampling restricts the representativeness of the findings. Future research should employ longitudinal designs, include more diverse samples, and incorporate multimethod assessments to better clarify the temporal and causal relationships between ON, transdiagnostic factors, and eating motivations.

## Conclusions

The current study highlights the close links between orthorexia nervosa, eating disorder symptomatology, perfectionism, and health-related motivations, while showing only weak associations with obsessive–compulsive symptoms. Difficulties in emotion regulation further contribute to the maladaptive aspects of ON. These findings suggest that orthorexia nervosa should be conceptualized as a multidimensional phenomenon shaped by both transdiagnostic traits and health-driven eating motives, with important implications for assessment, prevention, and intervention.

## Data Availability

The datasets generated during and/or analysed during the current study are available from the corresponding author on reasonable request.
